# A novel treatment strategy for bladder hypoplasia: A case of megaureter in a functional solitary kidney

**DOI:** 10.1002/iju5.12713

**Published:** 2024-02-25

**Authors:** Yutaro Sasaki, Masayuki Takahashi, Mitsuki Nishiyama, Saki Kobayashi, Yoshiteru Ueno, Junya Furukawa, Kenji Shimada

**Affiliations:** ^1^ Department of Urology Tokushima University Graduate School of Biomedical Sciences Tokushima Japan; ^2^ Department of Urology Hirakata General Hospital for Developmental Disorders Hirakata Japan

**Keywords:** bladder hypoplasia, functional solitary kidney, home bladder cycling, megaureter, obstructive renal failure

## Abstract

**Introduction:**

We report a case of megaureter in a functional solitary kidney in which surgery was performed after bladder capacity was increased by home bladder cycling.

**Case presentation:**

A 6‐day‐old girl with a left megaureter, a right multicystic dysplastic kidney, and bladder hypoplasia underwent percutaneous left nephrostomy for obstructive renal failure. At 8 months, home bladder cycling was initiated to increase bladder capacity before the planned ureterocystoneostomy. Surgery was performed after bladder capacity increased. The left ureter was compressed by the left umbilical ligament, so ureteral end‐to‐end anastomosis was performed at 1 year and 4 months. At 2 years and 8 months, cystometry showed age‐appropriate bladder capacity and improved bladder compliance.

**Conclusion:**

To the best of our knowledge, this is the first report of bladder hypoplasia treated by home bladder cycling.

**Abbreviation & Acronym**
VUR: vesicoureteral reflux

**Keynote message**

We report a case of megaureter in a functional solitary kidney in which surgery was performed after bladder capacity was increased by home bladder cycling. We propose a novel stepwise strategy for the treatment of megaureter in a functional solitary kidney that consists of urinary drainage followed by home bladder cycling and finally urinary reconstructive surgery.

## Introduction

In a patient with megaureter in a functional solitary kidney, the bladder cannot distend with urine, resulting in a hypoplastic bladder.^1^, ^2^ Only limited evidence is available on the treatment for bladder hypoplasia secondary to this malformation.^2^, ^3^, ^4^, ^5^ Here, we report a case of bladder hypoplasia in which a novel, simple, and minimally invasive treatment strategy was performed, including home bladder cycling.

## Case presentation

**Fig. 1:**
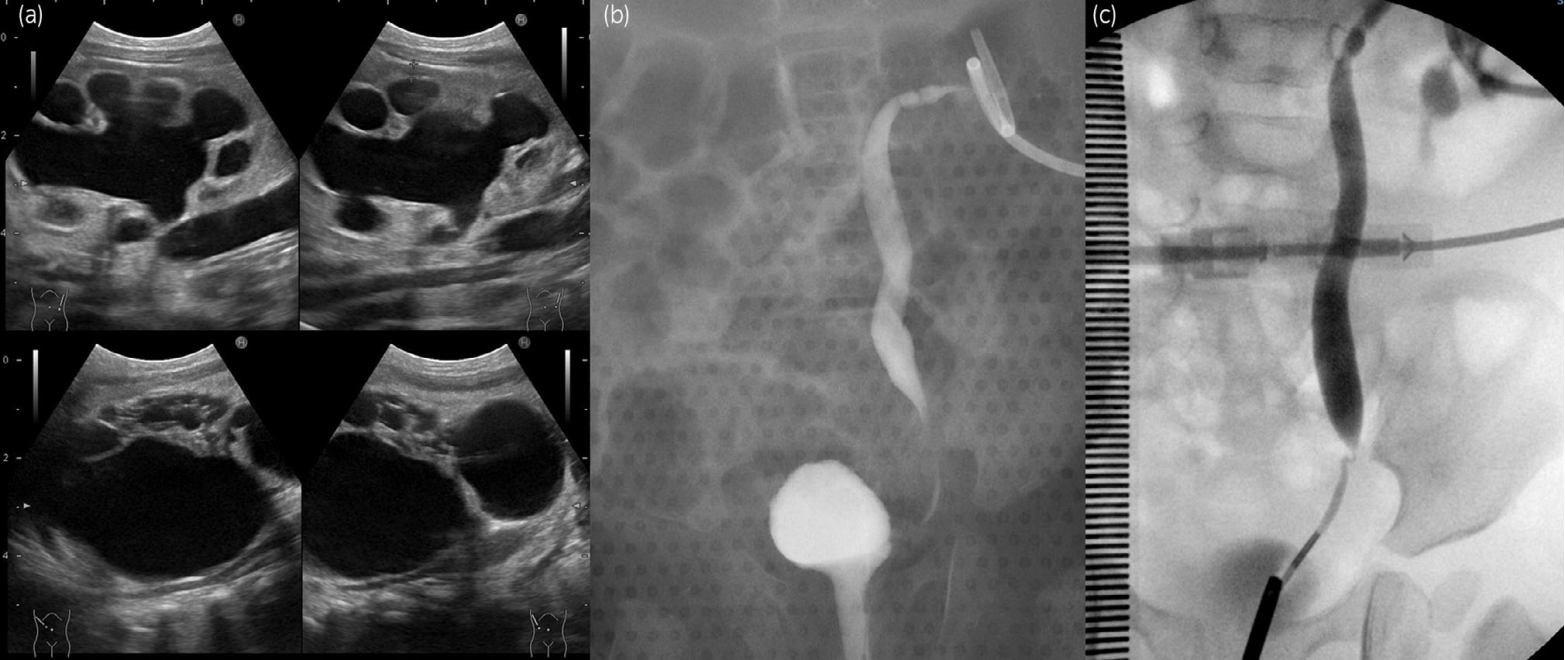
(a) Ultrasonographic images obtained soon after birth showing hydronephrosis grade 4 on the left, left megaureter (upper image), and a right multicystic dysplastic kidney (lower image). (b) A voiding cystourethrogram was obtained at 1 month showing left VUR grade 2 with a very small bladder. (c) Retrograde pyelography was performed at 1 year and 2 months. The site of the stricture was found to be 3 cm cranial from the bladder.

**Fig. 2:**
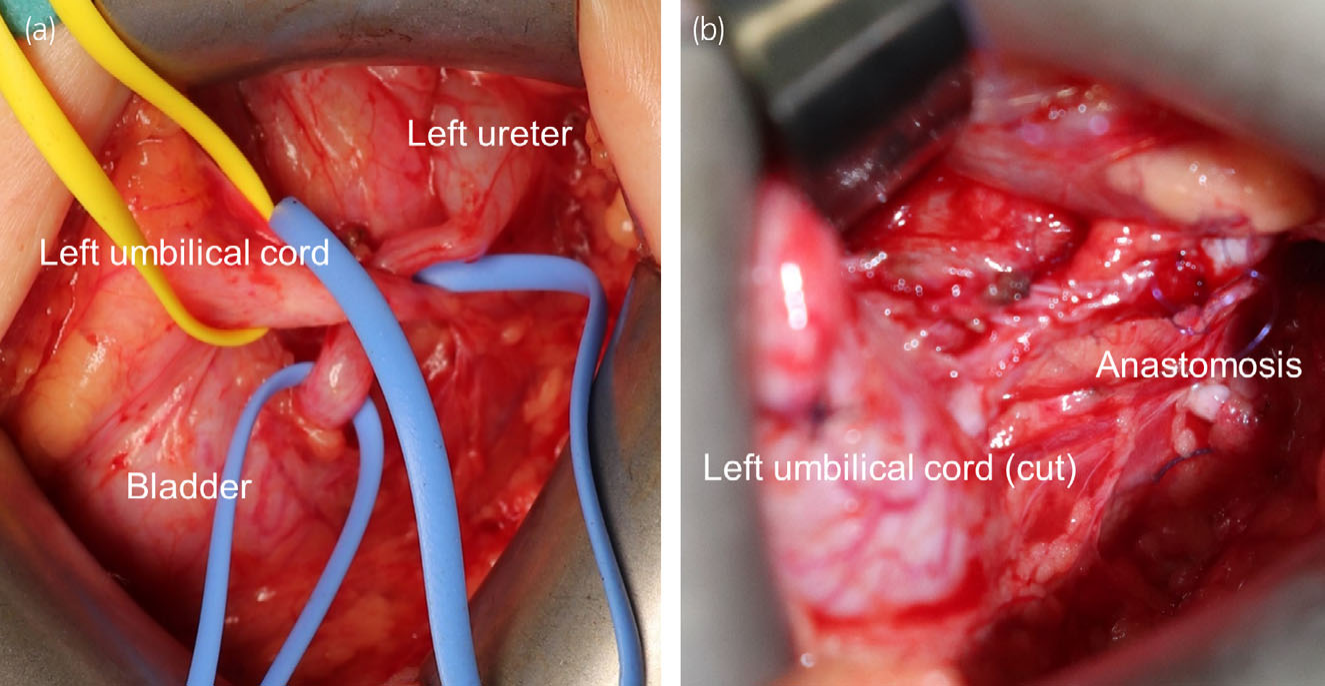
Images showing urinary reconstructive surgery. (a) The site of occlusion was compressed by the left umbilical cord. (b) The left umbilical cord was dissected and ligated with 4‐0 Vicryl. A left‐sided ureteral end‐to‐end anastomosis was performed using 6‐0 PDS BV‐1.

**Fig. 3:**
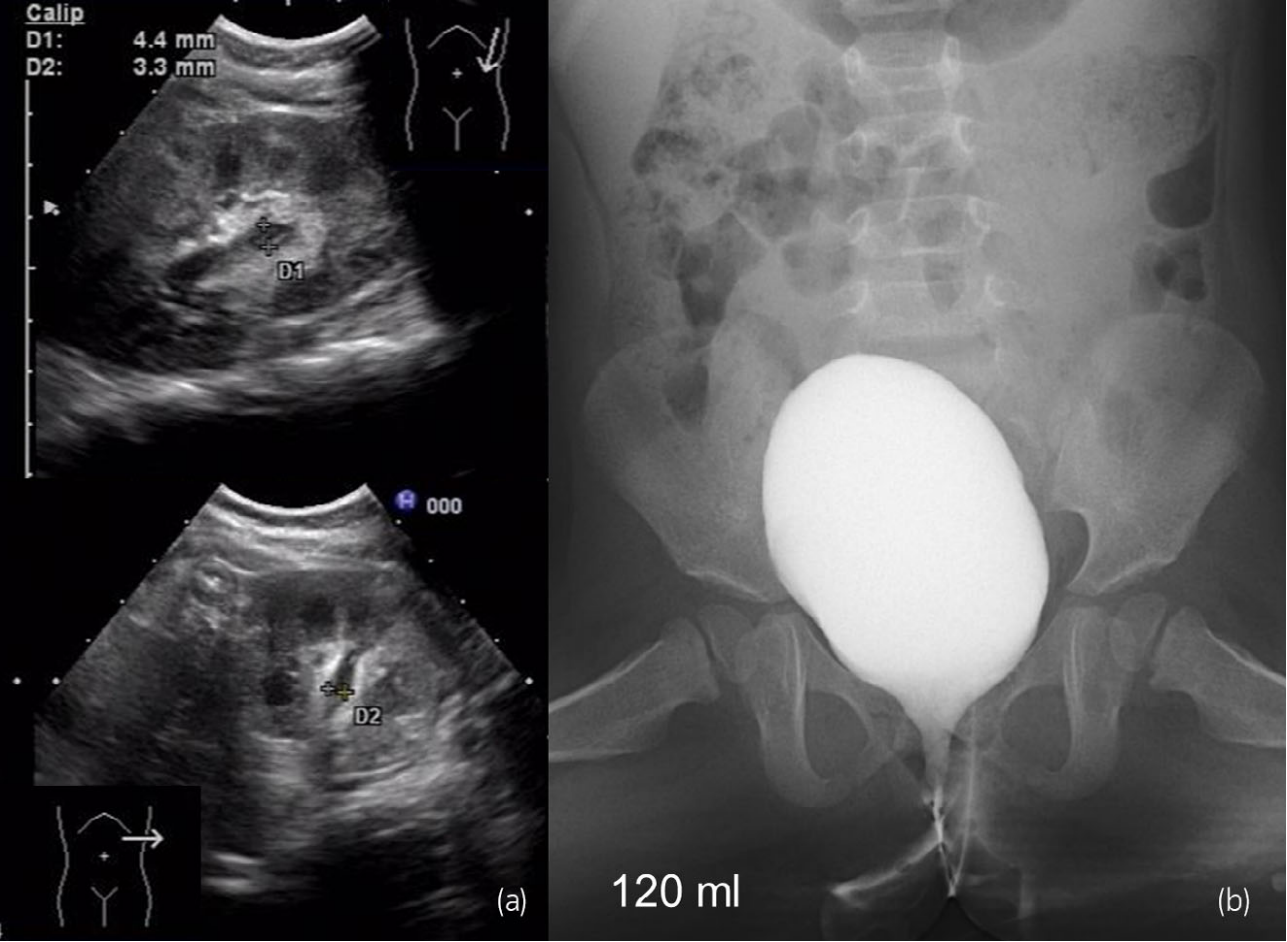
(a) Ultrasonographic images obtained at 2 years of age showing left‐sided hydronephrosis grade 1. (b) Voiding cystourethrography and cystometrometry performed at 2 years and 8 months show disappearance of VUR, improvement of bladder capacity to 120 mL, and improvement of bladder compliance to 13.3 mL/cmH_2_O.

A female neonate with a right multicystic dysplastic kidney and left hydronephrosis grade 3 on ultrasonography was born at 38 weeks and 6 days (vaginal delivery, 2976 g). Her mother had oligohydramnios during pregnancy. Ultrasonography was performed soon after birth revealed left hydronephrosis grade 4, left megaureter, and a right multicystic dysplastic kidney (Fig. 1a). Her bladder did not have the opportunity to distend with urine, resulting in bladder hypoplasia. She also developed obstructive renal failure (serum creatinine 0.92 mg/dL) and underwent percutaneous left nephrostomy on Day 6 after birth. Voiding cystourethrography at 1 month of age showed left VUR grade 2 (Fig. 1b). Furthermore, only 10 mL of contrast medium could be injected because of bladder hypoplasia. Initially, we opted for elective surgery while periodically replacing the nephrostomy catheter. At 8 months, home bladder cycling was initiated with the aim of increasing bladder capacity and was performed as follows. An 8‐Fr cuffed urinary catheter was placed by the parents. An uncuffed Nelaton catheter was found to be of no use because it dislodged rapidly with movement. Normal saline was dripped naturally into the bladder through the urinary catheter. The Bladder was exposed to a constant pressure of 20 cmH_2_O for 10 min by positioning a 500‐mL saline bag 20 cm above the bladder. Initially, most of the saline leaked from the side of the urethra, but slowly started to accumulate in the bladder. The amount of saline used for one bladder cycling was 50–150 mL. Home bladder cycling was performed three times weekly. There were no complications related to home bladder cycling. At 1 year and 2 months, cystography and cystometry showed a morphologically normal bladder; the capacity had increased to 70 mL but the bladder still showed low compliance (0.94 mL/cmH_2_O). Retrograde pyelography was performed at this time. The site of the stricture was found to be 3 cm cranial from the bladder (Fig. 1c). We considered the possibility of megaureter with a long narrow segment. At 1 year and 4 months, an open left ureteral end‐to‐end anastomosis was performed. We performed a 5.0‐cm Pfannenstiel incision to expose the bladder and left ureter. The site of the stricture was compressed by the left umbilical cord (Fig. 2a), which was dissected and ligated with 4‐0 Vicryl. A left ureteral end‐to‐end anastomosis was performed with 6‐0 PDS BV‐1 (Fig. 2b). The operation time was 193 min. Postoperatively, she was started on oral oxybutynin 0.2 mg/kg/day. The nephrostomy catheter was removed 4 months after surgery. At 2 years of age, her serum creatinine level was 0.28 mg/dL, and ultrasonography revealed left‐sided hydronephrosis grade 1 (Fig. 3a). At 2 years and 8 months, voiding cystourethrography and cystometrometry confirmed disappearance of VUR, improvement of bladder capacity to 120 mL, and improvement of bladder compliance to 13.3 mL/cmH_2_O (Fig. 3b). Oxybutynin was discontinued at this time. There have been no recurrences of urinary tract infection in the 2 years and 5 months since surgery.

## Discussion

Megaureter in a functional solitary kidney, bilateral single ectopic ureters, and a single ectopic ureter in a functional solitary kidney are rare urinary tract malformations that result in the bladder not being able to distend with urine and bladder hypoplasia.^1^, ^2^ Furthermore, megaureter in a functional solitary kidney may progress to obstructive renal failure because of poor drainage of urine. Therefore, a patient with this malformation requires urinary tract drainage, such as nephrostomy or cutaneous ureterostomy, immediately after birth. Our patient underwent percutaneous left‐sided nephrostomy for obstructive renal failure on Day 6 after birth as a life‐saving measure. Early urinary reconstructive surgery, such as ureteroneocystostomy, was not possible because of bladder hypoplasia. Urinary diversion was a treatment option, but was considered too invasive in this case. Therefore, we opted for elective surgery while periodically replacing the nephrostomy catheter. We also treated the bladder hypoplasia to increase bladder capacity for planned ureterocystoneostomy. There is limited information available on treatment of bladder hypoplasia secondary to this malformation.^2^, ^3^, ^4^, ^5^ Shimada et al. described two‐stage surgery for a single ectopic ureter in a patient with a solitary kidney and a hypoplastic bladder.^2^ Ureterovesicostomy between the dilated ureter and the lateral wall of the bladder was performed as a first‐stage surgery to increase bladder capacity. The patient in their report underwent ureteroneocystostomy as a second‐stage surgery after the bladder capacity had increased sufficiently for reimplantation. Although this two‐stage surgery was a breakthrough strategy, ureteral and bladder adhesions caused by the first‐stage surgery could make the second‐stage surgery difficult, especially for surgeons in non‐high‐volume centers. Yamamoto et al. have reported on the efficacy of double‐J stent placement instead of first‐stage surgery.^4^ Double‐J stent placement is minimally invasive, but bladder hypoplasia may make placement difficult. Furthermore, a patient with a double‐J stent may develop VUR, leading to recurrent febrile urinary tract infections that impair renal function. Infection‐related adhesions can complicate second‐stage surgery. Serrano et al. reported that bladder cycling can help refunctionalize the long‐term disused bladder before kidney transplantation.^6^ Based on this report, we performed home bladder cycling in this case. Home bladder cycling is simple, minimally invasive, and has the advantage of not affecting second‐stage surgery. In this case, we performed home bladder cycling using the cuffed urinary catheter for convenience. When performing this procedure on male patients, it is necessary to be aware of the increased risk of urethral injury. For male patients, it is safer to use an uncuffed Nelaton catheter. In this case, cystography performed 4 months after starting home bladder cycling revealed a morphologically normal bladder that had increased to a maximum capacity of 70 mL. Therefore, we determined that bladder capacity had increased sufficiently for ureteroneocystostomy. Intraoperatively, the site of obstruction was found to be compressed by the umbilical cord, so we performed a ureteral end‐to‐end anastomosis rather than ureteroneocystostomy. Although this treatment requires the cooperation of the patient's family, we believe that it is one of the best treatments for bladder hypoplasia in appropriately selected patients. Home bladder cycling allows the desired outcomes to be achieved without complicated reconstructive surgery and further morbidity.^5^

## Conclusion

To the best of our knowledge, this is the first report of bladder hypoplasia treated by home bladder cycling. We propose a novel stepwise strategy for the treatment of megaureter in a functional solitary kidney that consists of urinary drainage followed by home bladder cycling and finally urinary reconstructive surgery.

## Author contributions

Yutaro Sasaki: Project administration; writing – original draft. Masayuki Takahashi: Project administration; writing – review and editing. Mitsuki Nishiyama: Project administration. Saki Kobayashi: Project administration. Yoshiteru Ueno: Project administration. Junya Furukawa: Project administration; writing – review and editing. Kenji Shimada: Project administration; writing – review and editing.

## Conflict of interest

The authors declare no conflict of interest.

## Approval of the research protocol by an Institutional Reviewer Board

This research was conducted in accordance with the provisions of the Declaration of Helsinki.

## Informed consent

We obtained consent from the patient's parents for publication of this case report.

## Registry and the Registration No. of the study/trial

None.

## Animal studies

None.

## Funding information

This research did not receive any specific grant from funding agencies in the public, commercial, or not‐for‐profit sectors.
